# ﻿Three new species of *Gerronema* (Agaricales, Basidiomycota) from southern China

**DOI:** 10.3897/mycokeys.114.145299

**Published:** 2025-03-04

**Authors:** Wei-Xin Zhang, Wang-Qiu Deng, Chang-Qing Chang, Ping Zhou, Min Lin, Ming Zhang

**Affiliations:** 1 Guangdong Provincial Key Laboratory of Microbial Signals and Disease Control, Engineering Research Center of Biological Control, Ministry of Education, State Key Laboratory for Conservation and Utilization of Subtropical Agro-Bioresources, South China Agricultural University, Guangzhou 510642, China Institute of Microbiology, Guangdong Academy of Sciences Guangzhou China; 2 State Key Laboratory of Applied Microbiology Southern China, Guangdong Provincial Key Laboratory of Microbial Culture, Collection and Application, Institute of Microbiology, Guangdong Academy of Sciences, Guangzhou 510070, China South China Agricultural University Guangzhou China; 3 Guangdong Nanling Forest Ecosystem National Observation and Research Station, Guangzhou, China Guangdong Nanling Forest Ecosystem National Observation and Research Station Guangzhou China

**Keywords:** New taxa, phylogenetic analysis, Porotheleaceae, taxonomy

## Abstract

Three new species of *Gerronema* are discovered from southern China. *Gerronemaangustum* is characterized by its small basidiomata, greenish-green pileus, slender stipe, narrow, and close lamellae. *Gerronemapubescence* is characterized by its pubescent pileus when young, yellowish white to pale yellow lamellae that are lighter towards the margin, narrowly cylindrical to lageniform pleurocystidia, and absent cheilocystidia. *Gerronemarhizomorpha* is characterized by its yellowish white to brown pileus, well-developed rhizomorphs at the stipe base, absent cheilocystidia and pleurocystidia, and not growing on rotten wood. Molecular phylogenetic analyses of nrITS + nrLSU support the species delimitation. In this study, detailed descriptions, photos of the basidiomata, line drawings, and discussions with related species are comprehensively provided. A key to the known *Gerronema* species in China is also provided.

## ﻿Introduction

*Gerronema* Singer (Porotheleaceae, Agaricales, Agaricomycetes, Basidiomycota), typified by *G.melanomphax* Singer, was first proposed to accommodate the three “rebellious” species from Argentina, which were characterized by their somewhat thick-walled hyphae and consequently more elastic-toughish consistency, strongly irregular hymenophoral trama, strongly intracellular pigmentation, and lignicolous habitat (Singer 1951). In this case, species of *Gerronema* only differ from species in *Omphalina* Quél. by the absence of fuscous, intraparietal, or incrusting pigments (Singer 1964, 1986; Bigelow 1970, 1982, 1985). Lange (1981) suggested that the characters defining various segregates of *Omphalina* are hardly of sufficient significance on the generic level, and *Gerronema* was regarded as a subgenus of *Omphalina*. Later, the characteristics of *Gerronema* were redefined as basidiomata omphalinoid to clitocyboid, pileus convex to infundibuliform or umbilicate, lamellae decurrent and subdistant, stipe central, basidiospores thin-walled, smooth, inamyloid, tramal tissue sarcodimitic, occasionally with cystidia and clamp connections, and lignicolous habitat (Redhead 1986; Norvell et al. 1994). And *Chrysomphalinastrombodes* (Berk. & Mont.) Clémençon and *Clitocybexanthophylla* Bres. were transferred into *Gerronema* (Norvell et al. 1994). Up to now, this definition is widely accepted by some scholars (Bañares et al. 2006; Antonín et al. 2008; Latha et al. 2018; Liu et al. 2019; Jayawardena et al. 2022; Na et al. 2022, 2024).

In previous studies, *Gerronema* was considered to be heterogenous by some scholars (Clémençon 1982; Moser 1983; Kuyper 1986; Singer 1986; Norvell et al. 1994). However, some researchers regarded *Gerronema* as a monophyletic group as restricted by Norvell et al. (1994) and included into the hydropoid clade together with *Clitocybula* (Singer) Singer ex Métrod, *Hydropus* Kühner ex Singer, *Megacollybia* Kotl. & Pouzar, and *Porotheleum* Fr. (Norvell et al. 1994; Moncalvo et al. 2002; Antonín et al. 2008, 2019; Yang et al. 2012). Molecular phylogenetic analyses provided new perspectives, and *Gerronema* was proved to be a polyphyletic group (Lutzoni 1997; Moncalvo et al. 2002; Redhead et al. 2002; Latha et al. 2018). The genus was resolved into several clades and closely related to the genera *Megacollybia* and *Trogia* Fr. in the family Porotheleaceae (Vizzini et al. 2019; Na et al. 2022, 2024). Up to now, the phylogenetic systematic position of *Gerronema* remains unclear due to the insufficient number of specimens and the limitation of phylogenetic research progress.

*Gerronema* is a small genus; only 75 species names have been recorded in Index Fungorum (http://www.indexfungorum.org, 1 November 2024). Most species of *Gerronema* are distributed in subtropical to tropical regions (Singer 1970; Norvell et al. 1994). China is one of the countries with the highest biodiversity and rich species, but only twelve *Gerronema* species have been reported, namely, *G.albidum* (Fr.) Singer, *G.baisanzuens* Q. Na, H. Zeng & Y.P. Ge, *G.brunneosquamulosum* Q. Na & Y.P. Ge, *G.chrysocarpum* P.G. Liu, *G.confusum* L. Fan & T.Y. Zhao, *G.indigoticum* T. Bau & L.N. Liu, *G.lapidescens* (Horan.) Ming Zhang & W.X. Zhang, *G.kuruvense* K.P.D. Latha & Manim., *G.microcarpum* Q. Na, H. Zeng & Y.P. Ge, *G.nemorale* Har. Takah., *G.strombodes* (Berk. & Mont.) Singer and *G.zhujian* Q. Na, H. Zeng & Y.P. Ge, seven of them are originally described from China (Liu 1995; Liu et al. 2019; Dai et al. 2010; Li et al. 2021; Wu et al. 2021; Na et al. 2022, 2024; Zhao and Fan 2022; Zhang et al. 2024). During our field investigation in southern China, three new species of *Gerronema* were found; they were formally described and introduced in morphological characters with molecular data in the present study.

## ﻿Materials and methods

### ﻿Sample collection and morphological study

Macroscopic morphological characteristics were derived from observation records and color images of fresh specimens collected in the field. Color descriptions were obtained according to Kornerup and Wanscher (1978). Samples were dried using an electric dryer at 50 °C and then deposited in the Fungarium of the Institute of Microbiology, Guangdong Academy of Sciences, Guangzhou, China (GDGM). The specific operations of the microscopic morphological characteristics are as follows: Sample each tissue section with tweezers or blades, place it in the slide floating carrier (1 drop of 5% KOH), stain with 1% Congo red solution, and observe the microstructure and measure it with a light microscope (Nikon Ni-U, Nikon Corporation, Japan). Twenty mature spores and 10 basidia were selected for measurement and represented by (a)b–c(d), where a or d indicates the extreme values, and b–c contains 90% of the measurements. L, W, and Q refer to length, width, and L/W ratio, respectively; L_m_, W_m_, and Q_m_ refer to the mean length, width, and Q value of all basidiospore samples ± the standard deviation, respectively.

### ﻿DNA extraction, polymerase chain reaction (PCR) amplification, and sequencing

Genomic DNA samples were extracted from dried specimens using the HiPure Fungal DNA Kit (Magen Biotechnology Co., Ltd., Guangzhou, China) and kept in a -20 °C refrigerator. The internal transcribed spacer (nrITS) and the large subunit nuclear ribosomal DNA gene (nrLSU) were respectively amplified with primer pairs ITS1/ITS4 and LROR/LR7 (White et al. 1990; Hopple and Vilgalys 1999). PCR reactions were performed in a total volume of 25 μL containing 1 μL template DNA, 9.5 μL distilled water, 1 μL of each primer, and 12.5 μL 2 × PCR mix (DreamTaq^tm^ Green PCR Master Mix, Fermentas) (Zhang et al. 2022). The PCR procedure amplification was as follows: pre-denaturation at 95 °C for 5 min, 35 cycles of denaturation at 95 °C for 30 s, annealing at 56 °C (for nrITS)/50 °C (for nrLSU) for 40 s, extension at 72 °C for 50 s, and final extension at 72 °C for 10 min. After the amplification products were tested by agarose gel electrophoresis, PCR products were sent to Beijing BGI Co., Ltd. (Guangzhou, China) for sequencing. Then the sequences of the bidirectional sequencing were checked and assembled by the Geneious Pro trial 4.8.4 (Biomatters Limited Company). The newly obtained sequences were submitted to GenBank.

### ﻿Phylogenetic analyses

The nrITS and nrLSU datasets were concatenated, including newly generated sequences, some valuable *Gerronema* sequences, and related genera (*Clitocybula*, *Hydropus*, *Leucoinocybe* Singer ex Antonín, Borovička, Holec & Kolařík, *Marasmiellomycena* De la Peña-Lastra, Mateos, Kolařík, Ševčíková & Antonín, *Megacollybia*, *Porotheleum*, *Pseudohydropus* Vizzini & Consiglio, *Pulverulina* Matheny & K.W. Hughes, and *Trogia*) sequences in GenBank were selected for phylogenetic analyses based on previous studies (Na et al. 2022, 2024) and listed in Table 1. *Mycenapurpureofusca* (Peck) Sacc. was selected as the outgroup according to a previous study (Na et al. 2022). Using the auto strategy with MAFFT v7.505 (Katoh and Standley 2013) and then manually edited in MEGA v11.0.10 (Koichiro et al. 2021). The best model of nucleotide evolution for the dataset (nrITS + nrLSU) was identified using PartitionFinder 2 (Lanfear et al. 2016). Bayesian Inference (BI) and Maximum Likelihood (ML) bootstrap analyses were performed using the best-fit substitution models identified in PhyloSuite v1.2.3 (Zhang et al. 2020). The BI analysis was carried out in MrBayes 3.2.6 (Ronquist et al. 2012) under the best-fit substitution model, in which the initial 25% of sampled data were discarded as burn-in. Maximum likelihood phylogenies were inferred using IQ-TREE (Nguyen et al. 2015) under the edge-linked partition model for 5000 ultrafast (Minh et al. 2013) bootstraps. Phylogenetic trees were visualized using FigTree v.1.4.4. The maximum likelihood bootstrap over 50% (MLB ≥ 50%) and the Bayesian posterior probability over 0.90 (BPP ≥ 0.90) were shown.

**Table 1.** Information for the sequences used in the phylogenetic analyses. Newly generated sequences are in bold.

Taxon	Voucher	Locality	GenBank accession No.		Reference
			nrITS	nrLSU	
*Clitocybulaabundans*	STU:SMNS-B-FU-2017/00898	Germany	MF627833	–	Direct Submission
*C.familia*	PRM 921866	Czech Republic	JF730327	JF730320	Antonín et al. (2011)
*C.fuscostriata*	FFAAS1030	China	OR238882	OR238894	Na et al. (2024)
*Gerronemaalbidum*	H:6050710	USA	–	MF318923	Direct Submission
*G.albidum*	H:6059277	USA	–	MF318924	Direct Submission
***G.angustum***	**GDGM 88662**	**China**	**PQ452698**	**PQ350413**	**This study**
***G.angustum***	**GDGM 88663**	**China**	**PQ452699**	–	**This study**
*G.atrovirens*	BKF10264	Thailand	MZ452088	MZ452671	Jayawardena et al. (2022)
*G.atrovirens*	BKF10265	Thailand	MZ452668	MZ452672	Jayawardena et al. (2022)
*G.baishanzuense*	FFAAS0359	China	OL985962	OL985984	Na et al. (2022)
*G.baishanzuense*	FFAAS0360	China	OL985963	–	Na et al. (2022)
*G.baishanzuense*	FFAAS0361	China	OL985964	–	Na et al. (2022)
*G.baishanzuense*	FFAAS0362	China	OL985965	OL985986	Na et al. (2022)
*G.baishanzuense*	FFAAS0363	China	OL985966	OL985987	Na et al. (2022)
*G.baishanzuense*	FFAAS0366	China	OL985967	OL985988	Na et al. (2022)
*G.brunneosquamulosum*	FFAAS1032	China	OR238884	OR238896	Na et al. (2024)
*G.brunneosquamulosum*	FFAAS1033	China	OR238885	OR238897	Na et al. (2024)
*G.citrinum*	G7458	French	MN994795	–	Jaouen et al. (2019)
*G.citrinum*	G7785	French	MN994822	–	Jaouen et al. (2019)
*G.citrinum*	PC0713130	French	MN994747	–	Jaouen et al. (2019)
*G.citrinum*	PC0714037	French	MN994655	–	Jaouen et al. (2019)
*G.confusum*	BJTC FM1592	China	OK161262	–	Zhao and Fan (2022)
*G.confusum*	BJTC FM1624	China	OK161271	–	Zhao and Fan (2022)
*G.indigoticum*	HMJAU47636	China	MK693727	MK693732	Liu et al. (2019)
*G.indigoticum*	HMJAU47942	China	MK693728	MK693733	Liu et al. (2019)
*G.indigoticum*	HMJAU47943	China	MK693729	MK693734	Liu et al. (2019)
*G.keralense*	BKF10263	Thailand	MZ452107	MZ452144	Direct Submission
*G.keralense*	CAL 1666	India	MH156555	MH153979	Latha et al. (2018)
*G.kuruvense*	BKF10266	Thailand	MZ452090	MZ452669	Direct Submission
*G.kuruvense*	CAL 1665	India	MH156554	MH153978	Latha et al. (2018)
*G.kuruvense*	DCY3362(HGASMF01-15010)	China	MZ951144	–	Direct Submission
*G.kuruvense*	FCATAS9085	China	PP622159	–	Direct Submission
*G.kuruvense*	KUC20220701_03	Korea	OR600252	–	Cho et al. (2024)
*G.lapidescens*	GDGM 85271-1	China	OR736197	–	Zhang et al. (2024)
*G.lapidescens*	GDGM 85271-2	China	OR736198	–	Zhang et al. (2024)
*G.lapidescens*	GDGM 86705	China	OR736202	–	Zhang et al. (2024)
*G.microcarpum*	FFAAS0371	China	OL985968	OL985990	Na et al. (2022)
*G.microcarpum*	FFAAS0372	China	OL985969	OL985991	Na et al. (2022)
*G.microcarpum*	FFAAS0373	China	OL985970	OL985992	Na et al. (2022)
*G.microcarpum*	FFAAS0374	China	OL985971	–	Na et al. (2022)
*G.microcarpum*	FFAAS0375	China	OL985972	OL985993	Na et al. (2022)
*G.nemorale*	HMJAU59063	China	OK560883	–	Direct Submission
*G.nemorale*	HMJAU59064	China	OK560871	–	Direct Submission
*G.nemorale*	FA236	Pakistan	MN744687	–	Aqdus and Khalid (2021)
*G.nemorale*	FA239	Pakistan	MN744688	–	Aqdus and Khalid (2021)
*G.nemorale*	FA249	Pakistan	MN744686	–	Aqdus and Khalid (2021)
*G.nemorale*	FFAAS0389	China	OL985981	OL986002	Na et al.(2022)
*G.nemorale*	FFAAS0392	China	OL985982	OL986003	Na et al.(2022)
*G.nemorale*	FFAAS0410	China	OL985983	OL986004	Na et al.(2022)
***G.pubescence***	**GDGM 93936**	**China**	**PQ452700**	**PQ350414**	**This study**
***G.pubescence***	**GDGM 94001**	**China**	**PQ452701**	**PQ350415**	**This study**
***G.rhizomorpha***	**GDGM 87835**	**China**	**PQ452702**	–	**This study**
***G.rhizomorpha***	**GDGM 92067**	**China**	**PQ452703**	**PQ350416**	**This study**
*Gerronema* sp.	HMJAU59018	China	OK491123	–	Direct Submission
*G.strombodes*	FLAS-F-60957	USA	MH016911	–	Direct Submission
*G.strombodes*	FLAS-F-71339	USA	OR438652	–	Direct Submission
*G.strombodes*	TENN:F-60009	USA	KY271083	–	Direct Submission
*G.strombodes*	TFB12519/TENN60718	USA	EU623640	–	Hughes et al. (2007)
*G.strombodes*	TFB12783/TENN61350	USA	EU623641	–	Hughes et al. (2007)
*G.strombodes*	DJL05NC72	USA	EU623639	–	Hughes et al. (2007)
*G.subclavatum*	FLAS-F-60986	USA	MH016932	–	Direct Submission
*G.subclavatum*	FLAS-F-61518	USA	MH211945	–	Direct Submission
*G.subclavatum*	FLAS-F-71359	USA	OR242635	–	Direct Submission
*G.subclavatum*	iNaturalist # 8545787	India	MN906021	–	Direct Submission
*G.subclavatum*	Mushroom Observer # 243440	USA	MK607510	–	Direct Submission
*G.subclavatum*	S.D. Russell MycoMap # 6854	India	MN906138	–	Direct Submission
*G.subclavatum*	Smith-2018 iNaturalist # 17333993	USA	MK573888	–	Direct Submission
*G.viridilucens*	DED 7822	Brazil	–	OR449361	Direct Submission
*G.waikanaense*	PDD:87667	New Zealand	JQ694117	–	Direct Submission
*G.xanthophyllum*	PRM 924657	Czech Republic	LT854023	LT854023	Antonín et al. (2019)
*G.xanthophyllum*	SYKOf3970	Russia	OR915457	–	Direct Submission
*G.zhujian*	FFAAS0370	China	OL985974	OL985995	Na et al. (2022)
*G.zhujian*	FFAAS0376	China	OL985975	OL985996	Na et al. (2022)
*Hydropusfuliginarius*	S.D. Russell ONT iNaturalist # 130794969	USA	OP643427	–	Direct Submission
*H.marginellus*	OSC 112834	USA	EU669314	EU852808	Direct Submission
*H.rugosodiscus*	MGW1257	USA	KY777386	–	Direct Submission
*Leucoinocybedanxiashanensis*	GDGM 80184	China	MZ667478	MZ667482	Direct Submission
*L.lishuiensis*	FFAAS0115	China	MW424491	MW424495	Na et al. (2021)
*L.subglobispora*	FFAAS1034	China	OR238886	OR238898	Na et al. (2024)
*Oudemansiella* aff.	platyphylla 360-630	Japan	AB509870	–	Direct Submission
*Marasmiellomycenapseudoomphaliiformis*	BRNM:552721	USA	OR913562	OR913566	Senanayake et al. (2023)
*M.tomentosa*	FFAAS1036	China	OR238888	OR238900	Na et al. (2024)
*Megacollybiaclitocyboidea*	TENN62231	USA	EU623664	–	Hughes et al. (2007)
*M.marginata*	HR 91607	Czech Republic	LT854051	–	Antonín et al. (2019)
*M.platyphylla*	BRNM 737654	Czech Republic	LT854048	LT854036	Antonín et al. (2019)
*Mycenapurpureofusca*	HMJAU43554	China	MG654740	MK629356	Na and Bau (2018)
*M.purpureofusca*	HMJAU43624	China	MG654741	MK629357	Na and Bau (2018)
*Porotheleumfimbriatum*	CLZhao 1120	China	MH114870	–	Direct Submission
*P.fimbriatum*	Dai 12276	China	KX081137	KX161656	Direct Submission
*Pseudohydropusfloccipes*	BRNM 816173	Czech Republic	OM422758	OM423634	Direct Submission
*P.floccipes*	BRNM 825631	Spain	OM422760	OM423636	Consiglio et al. (2022)
*P.globosporus*	BAP 661	USA	MH414566	MH385340	Cooper et al. (2019)
*Pulverulinaflavoalba*	FFAAS1039	China	OR238891	OR238903	Na et al. (2024)
*P.flavoalba*	FFAAS1040	China	OR238892	OR238904	Na et al. (2024)
*P.ulmicola*	TFB13871	USA	MT237476	MT237446	Matheny et al. (2020)
*Trogiabenghalensis*	CUH AM031	India	KU647630	–	Dutta et al. (2017)
*T.infundibuliformis*	KUN_HKAS56709	China	JQ031776	JQ031781	Yang et al. (2012)
*T.venenata*	KUN_HKAS54710	China	JQ031772	JQ031778	Yang et al. (2012)

## ﻿Results

### ﻿Molecular phylogenetic results

**Figure 1.**
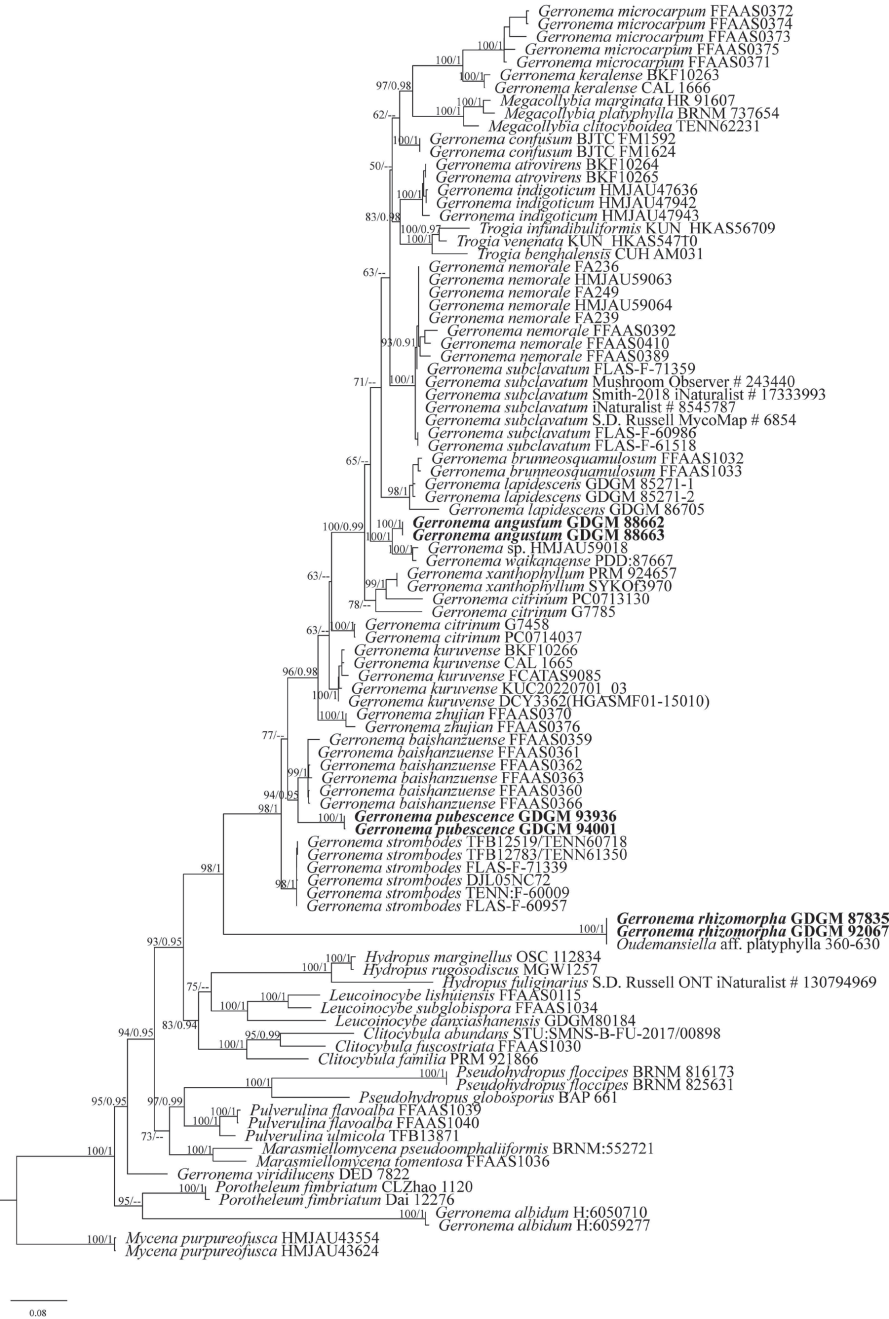
Phylogenetic consensus tree of *Gerronema* species inferred from the maximum likelihood and Bayesian inference based on a concatenated nrITS and nrLSU (MLB ≥ 50%, BPP ≥ 0.90 are shown). The tree is rooted with *Mycenapurpureofusca*. New taxa are shown in bold.

The final concatenated dataset consisted of 95 nrITS and 52 nrLSU sequences from 46 taxa of 11 genera of Physalacriaceae, Porotheleaceae, and Mycenaceae, which comprised 1927 nucleotide sites (942 for nrITS, 985 for nrLSU), of which 752 were parsimony-informative, 277 were singleton sites, and 898 were constant sites. For the ML analyses, the best-fit substitution models selected for nrITS and nrLSU region partitions in the concatenated dataset were HKY+I+G and GTR+I+G, respectively. For the BI analysis, the best-fit substitution model selected for each of the two DNA regions was GTR+I+G (2 parallel runs, 2000000 generations), and the average standard deviation of split frequencies was stably dropped under 0.01. The phylogenetic trees generated from BI and ML analyses show almost similar topologies and few variations in statistical support, so only the ML tree is displayed (Fig. 1).

In the phylogenetic tree (Fig. 1), *Clitocybula*, *Gerronema*, *Hydropus*, *Leucoinocybe*, *Marasmiellomycena*, *Megacollybia*, *Porotheleum*, *Pseudohydropus*, *Pulverulina*, and *Trogia* were nested in the core clade of Porotheleace with significant support (MLB = 100%, BPP = 1.00). The proposed new species formed three independent lineages within the genus *Gerronema* (MLB = 98%, BPP = 1.00), named *G.angustum*, *G.pubescence*, and *G.rhizomorpha*. In addition, *G.angustum* is sister to *G.waikanaense* (G. Stev.) J.A. Cooper and an unnamed specimen (HMJAU59018) (MLB = 100%, BPP = 1.00). *Gerronemapubescence* closely related to *G.baishanzuense* with a well-supported (MLB = 94%, BPP = 0.95). *Gerronemarhizomorpha* is placed at the base of the *Gerronoma* clade.

### ﻿Taxonomy

#### ﻿ Gerronema angustum

Taxon classificationFungiAgaricalesPorotheleaceae

Ming Zhang & W.X. Zhang sp. nov.

8DA9E13C-F8C6-5C8D-A347-650959A034E6

Fungal Names: FN 572081

Figs 2 , 3

##### Diagnosis.

Distinguished from other *Gerronema* species by the combination characters, including caespitose habit, greenish green pileus, narrow and close lamellae, slender stipe, baidiospores measuring (4)4.5–5.5 × 2.5–3.5 μm, mainly clavate to narrowly utriform cheilocystidia.

**Figure 2.**
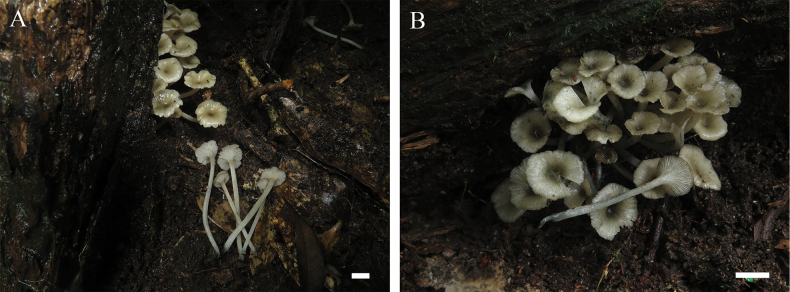
Basidiomata of *Gerronemaangustum***A** collection GDGM 88662, holotype **B** collection GDGM 88663. Photographed by Bin Song. Scale bars: 10 mm.

##### Holotype.

China • Guangdong Province: Shaoguan City, Nanling National Forest Park; 24°53'54"N, 113°2'24"E; 210 m asl.; 7 July 2022; Bin Song, Guo-Rui Zhong, and De-Chun Xie (GDGM 88662).

**Figure 3.**
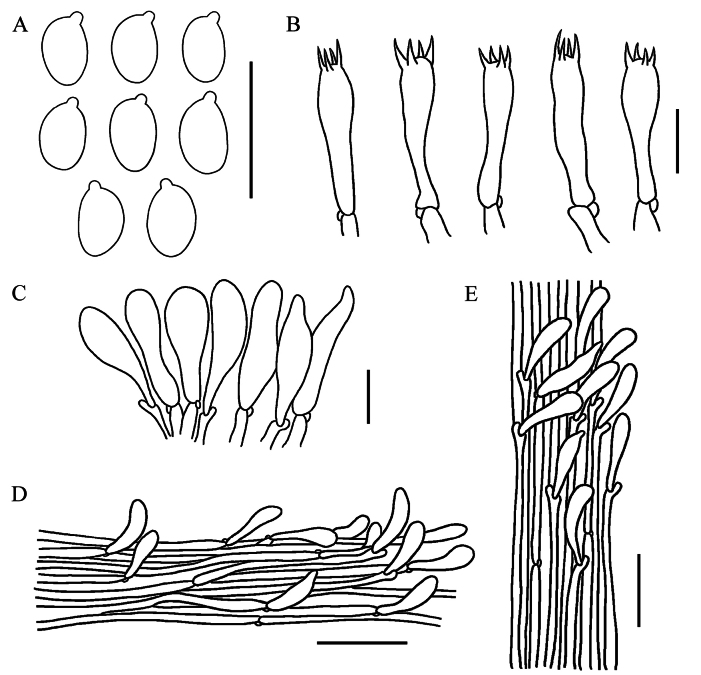
Microscopic features of *Gerronemaangustum* (GDGM 88662, holotype) **A** Basidiospores **B** Basidia **C** Cheilocystidia **D** Pileipellis **E** Stipitipellis. Scale bars: 10 μm (**A–C**); 50 μm (**D–E**).

##### Etymology.

*angustum* (Latin), referring to the narrow lamellae of this species.

##### Description.

Basidiomata small-sized. Pileus 10–18 mm broad, infundibuliform, umbilicate to deeply umbilicate at center, greyish brown to brown (6E3–4) when young, greyish green (30B3–4, 30C2–3) when old, greyish green (30E5–6) at center, surface moist, glabrous, margin inflexed, radially striped with greenish grey to dull green (30B4–5, 30D4–5) lines. Lamellae decurrent, close, narrow, arcuate, even, white (30A1) to greenish grey (30B2), with 1–3 lamellulae. Stipe 45–60 × 2–5 mm, slender, centric, cylindrical, hollow, fragile, grey to greyish green (30C1–3), covered with white (30A1) fibrils. Odor and taste not recorded.

Basidiospores (4)4.5–5.5 × 2.5–3.5 μm, L_m_ = 4.88 ± 0.51 µm, W_m_ = 2.96 ± 0.32 µm, Q = (1.33)1.43–1.83, Q_m_ = 1.66 ± 0.18, ellipsoid to oblong, smooth, thin-walled, hyaline, guttulate, inamyloid. Basidia 18–26 × 5.5–7 μm, clavate, thin-walled, hyaline, 4-spored, with sterigmata 2.3–4.4 µm long. Cheilocystidia 26–45 × 6–9.5 μm, clavate, fusiform to narrowly utriform, thin-walled, hyaline. Pleurocystidia not seen. Lamellar trama regular to subregular, hyphae 3–22 μm wide, cylindrical, thin-walled, hyaline. Pileipellis a cutis, hyphae 1.5–24.5 μm wide, smooth, hyaline; pileocystidia 22.5–65 × 8–15.5 µm, oblong to utriform, thin-walled, greyish brown to light brown pigmented in KOH. Pileus trama subregular, sarcodimitic. Stipitipellis a cutis, hyphae 3.5–25 μm wide, sometimes upturned hyphae, smooth, thin-walled, hyaline; caulocystidia 56–72 × 10.5–20.5 µm, narrowly cylindrical to oblong, thin-walled, hyaline. Stipe trama regular, sarcodimitic. Clamp connections present in all tissues.

##### Habit and distribution.

Caespitose on the rotten wood in broad-leaved forests. Currently only known from the type locality in China.

##### Additional specimen examined.

China • Guangdong Province: Shaoguan City, Nanling National Forest Park; 24°55'39"N, 113°3'20"E; 225 m asl.; 7 July 2022; Bin Song, Guo-Rui Zhong, and De-Chun Xie (GDGM 88663).

#### ﻿ Gerronema pubescence

Taxon classificationFungiAgaricalesPorotheleaceae

Ming Zhang & W.X. Zhang sp. nov.

FA946C68-3344-57E0-8C00-288F50B9D336

Fungal Names: FN 572082

Figs 4 , 5

##### Diagnosis.

Distinguished from other *Gerronema* species by the combination characters of the pastel grey pileus covered with pubescence when young, yellowish white to pale yellow lamellae are paler towards the margin, the absence of cheilocystidia, and the narrow cylindrical to utriform pleurocystidia.

**Figure 4.**
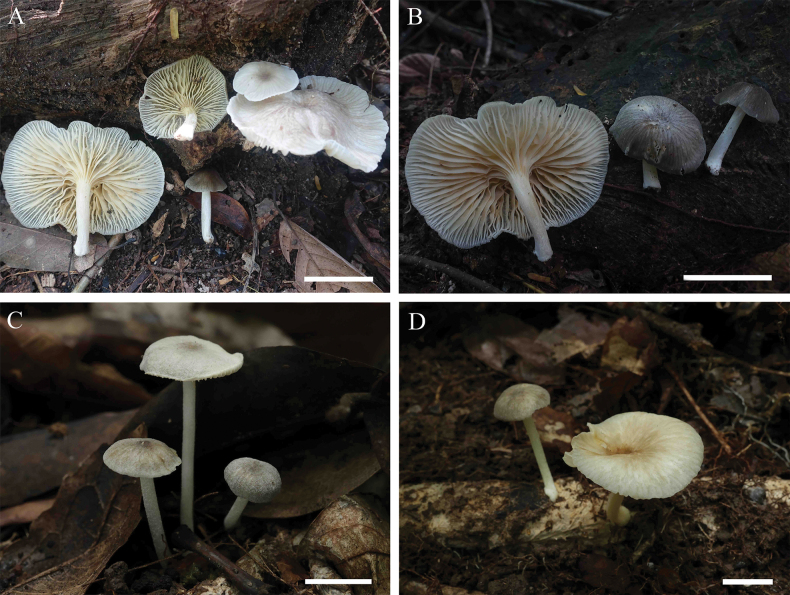
Basidiomata of *Gerronemapubescence***A, B** collection GDGM 94001, holotype **C, D** collection GDGM 93936. **A, B** Photographed by Hao Huang **C, D** photographed by Ming Zhang. Scale bars: 30 mm (**A, B**); 10 mm (**C, D**).

##### Holotype.

China • Guangdong Province: Huizhou City, Xiangtou Mountain Nature Reserve; 23°26'N, 114°37'E; 335 m asl.; 19 September 2023; Hao Huang and Wei-Xin Zhang (GDGM 94001).

**Figure 5.**
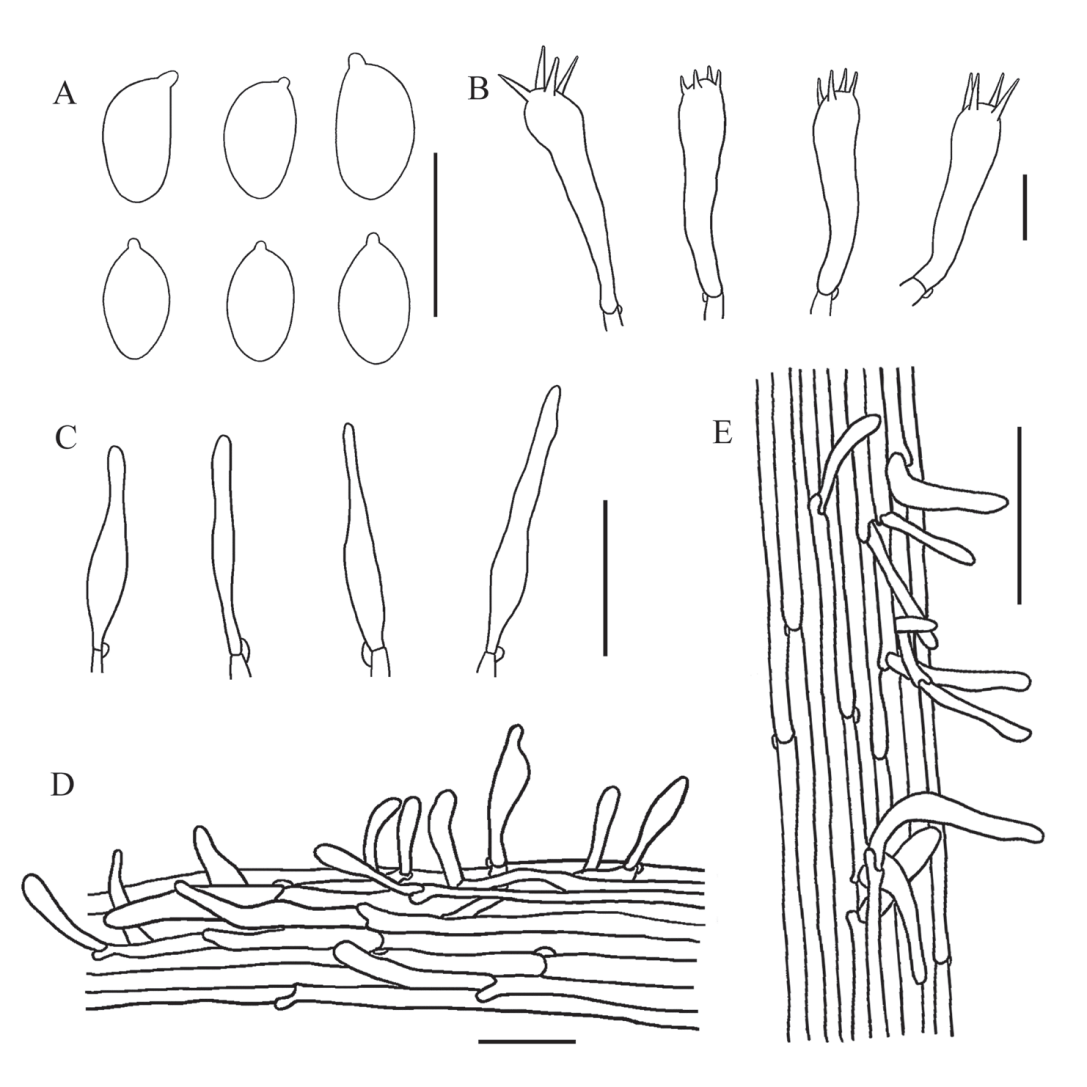
Microscopic features of *Gerronemapubescence* (GDGM 94001, holotype) **A** Basidiospores **B** Basidia **C** Pleurocystidia **D** Pileipellis **E** Stipitipellis. Scale bars: 10 mm (**A, B**); 50 mm (**C–E**).

##### Etymology.

*pubescence* (Latin), referring to the species pileus usually covered with pubescence when young.

##### Description.

Pileus 12–70 mm broad, hemispherical to plano-convex, depressed at center, grey (1E1), covered with pubescence when young, white to yellowish white (1A1–2) with age, grey to greyish brown (5E2–3, 6C1–2) at center, shallowly sulcate, surface dry, glabrous or pubescent, distinctly radially striped with grey to brownish orange (4C1–2, 6C2–3) lines, margin inflexed to reflexed. Lamellae subdecurrent, subdistant, ventricose, even, yellowish white to pale yellow (4A2–3), white (4C1) towards margin, with 1–5 lamellulae. Stipe 15–40 × 2–7 mm, central, cylindrical, hollow, white (1A1) to grey (1C1), covered with white granulose or fibrils. Odor and taste not recorded.

Basidiospores (6)6.5–8 × (3.5)4–4.5 μm, L_m_ = 7.13 ± 0.57 µm, W_m_ = 4.08 ± 0.29 µm, Q = (1.5)1.56–2, Qm = 1.75 ± 0.18, ellipsoid to oblong, smooth, thin-walled, hyaline, guttulate, inamyloid. Basidia 24.5–39 × 5–7.5 μm, clavate, thin-walled, hyaline, 2- or 4-spored, with sterigmata 2.5–5 µm long. Cheilocystidia absent. Pleurocystidia 40–104 × 8.5–12.5 µm, narrowly cylindrical to lageniform, thin-walled, hyaline. Lamellar trama regular to subregular, hyphae 3.5–26.5 μm wide, thin-walled, hyaline. Pileipellis a cutis, hyphae 3–25 μm wide, smooth, hyaline; pileocystidia 39–100 × 10–21 μm, oblong to narrowly clavate, apex sometimes rostrate, thin-walled, greyish brown pigmented in KOH. Pileus trama regular to subregular, sarcodimitic. Stipitipellis a cutis, hyphae 2.5–38.5 μm wide, smooth, thin-walled, hyaline; caulocystidia 38.5–84.5 × 8.5–19 μm, narrowly cylindrical to clavate, thin-walled, hyaline. Stipe trama regular, sarcodimitic. Clamp connections present in all tissues.

##### Habit and distribution.

Scattered or caespitose on the rotten wood in broad-leaved forests. Currently only known from the type locality in China.

##### Additional specimen examined.

China • Guangdong Province: Zhaoqing City, Dinghu Mountain Nature Reserve; 23°10'43"N, 112°33'10"E; 150 m asl.; 9 April 2024; Ming Zhang, Guo-Rui Zhong, and Wen-Xiao Xia (GDGM 93936).

#### ﻿ Gerronema rhizomorpha

Taxon classificationFungiAgaricalesPorotheleaceae

Ming Zhang & W.X. Zhang sp. nov.

DB0A81D2-3AFE-53D0-8D19-B4497941B821

Fungal Names: FN 572083

Figs 6 , 7

##### Diagnosis.

Distinguished from other *Gerronema* species by the combination characters of medium-sized basidiomata, yellowish white to brown pileus, well-developed rhizomorphs at stipe base, the absence of cheilocystidia, and pleurocystidia.

**Figure 6.**
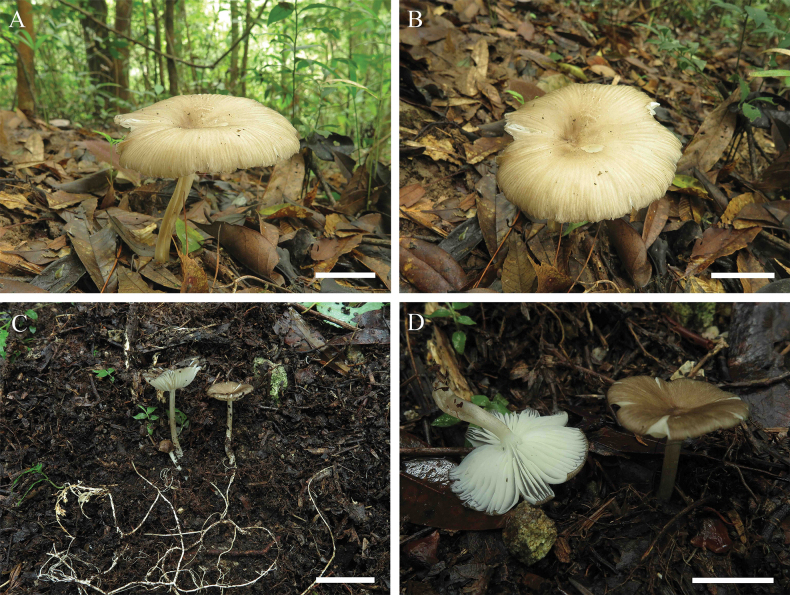
Basidiomata of *Gerronemarhizomorpha***A, B** collection GDGM 92067, holotype **C, D** collection GDGM 87835. Photographed by Ming Zhang. Scale bars: 30 mm (**A, B**); 50 mm (**C**); 30 mm (**D**).

##### Holotype.

China • Guangdong Province: Fengkai County, Zhaoqing City, Heishiding provincial natural reserve; 23°26'30"N, 111°53'28"E; 340 m asl.; 25 May 2023; Ming Zhang and Guo-Rui Zhong (GDGM 92067).

**Figure 7.**
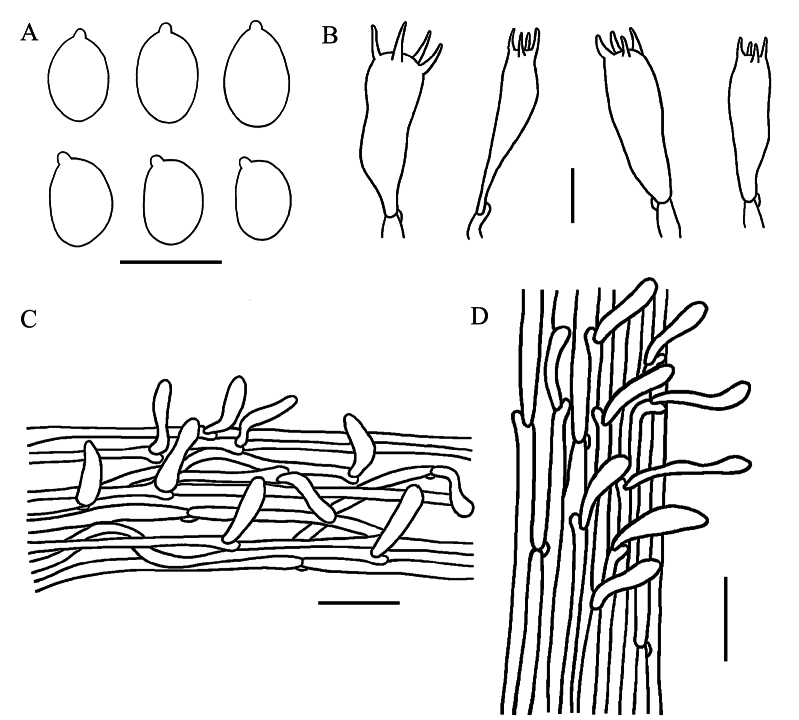
Microscopic features of *Gerronemarhizomorpha* (GDGM 92067, holotype) **A** Basidiospores **B** Basidia **C** Pileipellis **D** Stipitipellis. Scale bars: 10 mm (**A, B**); 50 mm (**C, D**).

##### Etymology.

*rhizomorpha* (Latin), referring to the species, usually has long and well-developed rhizomorphs at stipe base.

##### Description.

Basidiomata medium-sized. Pileus 35–95 mm broad, hemispherical to plano-convex, depressed at center, brown (5E5–6) when young, yellowish white (2A2–3) to brown (5E4–5) at maturity, usually brown (5E4–5) to dark brown (6F4–5) at center, surface dry, distinctly radially striped with brown (5E6–7, 6E4–5) lines, margin inflexed. Lamellae subdecurrent, subdistant, ventricose, even, white to yellowish white (1A1–2), with 1–3 lamellulae. Stipe 40–110 × 4–10 mm, central to eccentric, cylindrical, hollow, white to yellowish white (1A1–2), sometimes yellowish grey to greyish yellow (2C2–3) at the base, covered with yellowish grey to greyish yellow (2C2–4) fibrils, base with developed and white (1A1) rhizomorphs. Odor and taste not recorded.

Basidiospores 7–8.5(9.5) × 5–6(6.5) μm, L_m_ = 7.80 ± 0.71 µm, W_m_ = 5.53 ± 0.42 µm, Q = (1.23)1.27–1.58(1.6), Q_m_ = 1.41 ± 0.13, broadly ellipsoid to ellipsoid, smooth, thin-walled, hyaline, guttulate, inamyloid. Basidia 25.5–32 × 7.5–11.5 μm, clavate, thin-walled, hyaline, 4-spored, with sterigmata 2.8–6 µm long. Hymenial cystidia absent. Lamellar trama regular to subregular, hyphae 3–22 μm wide, thin-walled, hyaline. Pileipellis a cutis, hyphae 3–25 μm wide, thin-walled, light yellow in KOH; pileocystidia 25.5–63.5 × 6–14 μm, narrowly utriform to cylindrical, thin-walled, greyish brown to light brown pigmented in KOH. Pileus trama subregular, sarcodimitic. Stipitipellis a cutis, hyphae 1.5–11 μm wide, smooth, thin-walled, hyaline; caulocy stidia 12.5–33 × 3.5–8 μm, narrowly cylindrical to oblong, thin-walled, hyaline. Stipe trama regular, sarcodimitic. Clamp connections present in all tissues.

##### Habit and distribution.

Solitary or scattered, growing on the damp soil under broad-leaved forests. Currently known from China and Japan.

##### Additional specimen examined.

China • Guangdong Province: Shaoguan City, Nanling National Forest Park; 24°56'48"N, 113°3'19"E; 490 m asl.; 9 June 2022; Ming Zhang, Guo-Rui Zhong, and Shi-Zheng Wang (GDGM 87835).

## ﻿Discussion

Morphologically, *Gerronemaangustum* can be easily distinguished from other species in *Gerronema* by its caespitose habit, slender basidiomata, greenish-green pileus, narrow and close lamellae, and ellipsoid to oblong basidiospores measuring (4)4.5–5.5 × 2.5–3.5 μm. *Gerronemaangustum* is similar to *G.albidum*, which has been recorded in China (Singer 1962). But the distinctly white basidiomata and the absence of cheilocystidia can be used to distinguish *G.albidum* from *G.angustum*.

*Gerronemapubescence* is characterized by its hemispherical to plano-convex pileus covered with pubescence when young, yellowish white to pale yellow lamellae paler towards the pileus margin, absent cheilocystidia, and narrowly cylindrical to utriform pleurocystidia. *Gerronemapubescence* is similar to *G.keralense* K.P.D. Latha & Manim. and *G.zhujian*. However, *G.keralense*, originally described from India, can be distinguished by its small yellowish-brown pileus (4–17 mm broad), greyish-yellow stipe gradually greyish-brown towards the base, flexuose or irregular cheilocystidia, and absent pleurocystidia (Latha et al. 2018). *Gerronemazhujian* can be distinguished by the greyish-white pileus with a brown tinge at the center, slightly brown and narrow stipe (19–25 × 1.0–1.5 mm), subfusiform cheilocystidia, and the absence of pleurocystidia (Na et al. 2022).

*Gerronemarhizomorpha* is mainly characterized by its yellowish white to brown pileus, well-developed rhizomorphs at stipe base, and absent cheilocystidia and pleurocystidia. *Gerronemarhizomorpha* is similar to *G.confusum* in some extent as sharing relatively large basidiomata and brown pileus. But *G.confusum* from the north of China can be distinguished by its lignicolous habitat, greyish-brown stipe surface covered with dark brown granulose, absent rhizomorphs, 1–2-spored basidia, and abundant subcylindrical to cylindrical cheilocystidia (Zhao and Fan 2022). *Gerronemarhizomorpha* is also similar to *G.atrialbum* (Murrill) Borovička & Kolařík, with the stipe base often with rhizomorphs, white lamellae, and the absence of cheilocystidia and pleurocystidia. However, *G.atrialbum*, which is originally described from the USA, can be distinguished by its greyish brown to grey pileus and brown to pale stipe, relatively longer basidia (36–50 × 6.5–9.5 μm), which mainly has 2-spored. In addition, *G.atrialbum* mainly grows on humus or rotting hardwood (Murrill 1913; Antonín et al. 2019).

Phylogenetically (Fig. 1), our new species formed three distinct lineages according to the ML and BI phylogenetic analyses of the concatenated dataset and can be easily distinguished from other species with known sequences. *Gerronemaangustum* formed a distinct lineage in *Gerronema* and is sister to *G.waikanaense* with high statistical support (MLB = 100%, BPP = 1.00). However, *G.waikanaense*, reported from New Zealand, differs by its dark leaden grey basidiomata, minutely fibrillose stipe surface, distant lamellae, and absent cheilocystidia (Cooper 2014).

*Gerronemapubescence* is phylogenetically related to *G.baishanzuense* (MLB = 94%, BPP = 0.95). But *G.baishanzuense* can be distinguished by its relatively small pileus (3–25.5 mm broad), relatively short stipe (4.5–26 mm) densely covered with pruinose when young, clavate or subfusiform cheilocystidia usually swollen at apex, and absent pleurocystidia (Na et al. 2022).

*Gerronemarhizomorpha* was gathered together with an ITS sequence (AB509870) named *Oudemansiella* sp. (platyphylla 360–630) from Japan (MLB = 100%, BPP = 1.00) and shows that they represent the same phylogenetic species. It is noteworthy that *G.rhizomorpha* with well-developed rhizomorphs at stipe base and not growing on rotten wood is rare in *Gerronema*, which is morphologically more similar to species of the genus *Megacollybia*, but it does not belong to *Megacollybia* in phylogenetic analyses. Additionally, *G.rhizomorpha* has significant variability in the ITS1 region, has a low similarity rate with *Gerronema*, and is placed at the base of the *Gerronema* clade, possibly representing a separate evolutionary lineage. As the *Gerronema* genus is not a monophyletic group, *G.rhizomorpha* was temporarily classified as a member of *Gerronema* in the present study.

Due to *Gerronema* being widely distributed and many species being misidentified, only ten of the 20 species reported in Asia are from China. Recent investigations have found a high species diversity of *Gerronema* in southern China, and there are still many other species waiting to be reported, which can enrich the species diversity of the genus. In addition, the intraspecific and intergeneric phylogenetic relationships of *Gerronema* still remain highly controversial. Upon defining the diversity of species explicitly, the systematic phylogenetic framework of the genus *Gerronema* needs to be further refined based on more samples and sequence fragments to solve the classification problem of the *Gerronema*.

### ﻿Key to species of *Gerronema* in China

**Table:** 

1	Baisidiomata grow on soil, stipe base with well-developed rhizomorphs	**2**
–	Baisidiomata grow on decaying woods, stipe base without developed rhizomorphs	**3**
2	Sclerotia can be found at the base	***G.lapidescens***
–	Sclerotia not be found or recorded	***G.rhizomorpha***
3	Basidiomata blue	***G.indigoticum***
–	Basidiomata without blue tinge	**4**
4	Basidiomata greyish green	***G.angustum***
–	Basidiomata without greyish green tinge	**5**
5	Basidiomata white	***G.albidum***
–	Basidiomata without white tinge	**6**
6	Pleurocystidia present	**7**
–	Pleurocystidia absent	**8**
7	Pileus viscid	***G.chrysocarpum***
–	Pileus dry, usually covered with pubescence when young.	***G.pubescence***
8	Pileus densely covered with deep brown fur or scales	**9**
–	Pileus without fur or scales	**10**
9	Stipe fuscous, basidiospores 9.0–12.9 × 4.9–7.2 μm	***G.brunneosquamulosum***
–	Stipe white, basidiospores 6.3–8.5 × 3.2–4.8 μm	***G.zhujian***
10	Basidiomata small-sized, Pileus usually ≤ 11 mm in diam.	**11**
–	Basidiomata small to medium-sized, Pileus ≥ 11 mm in diam	**12**
11	Basidiospores 6.1–7.5 × 3.5–4.3 μm, cheilocystidia present	***G.microcarpum***
–	Basidiospores 8–11 × 4–6 μm, cheilocystidia absent	***G.kuruvense***
12	Basidia 1–2-spored	***G.confusum***
–	Basidia 4-spored	**13**
13	Pileus up to 25 mm in diam, stipe 4.5–26.0 × 0.5–2.0 mm	***G.baishanzuense***
–	Pileus less than 20 mm in diam, stipe 19.0–36.0 × 1.0–2.5 mm	***G.nemorale***

## Supplementary Material

XML Treatment for Gerronema angustum

XML Treatment for Gerronema pubescence

XML Treatment for Gerronema rhizomorpha

## ﻿Additional information

### Author ORCIDs

Ming Zhang https://orcid.org/0000-0001-6420-2531

### Data availability

All of the data that support the findings of this study are available in the main text.
